# Alleged Cruelty of a Hospital Sister

**Published:** 1886-11-20

**Authors:** 


### Alleged Cruelty of a Hospital Sister.

We received, a short time ago, a private communication from a highly respectable source, in which a "Sister," who occupies a position of responsibility at one of the best known London hospitals, was said to have behaved with great cruelty towards some of the patients. To such lengths, it was said, had the sister's cruelty been carried, that one poor patient, in despair of any redress, had attempted to commit suicide. This was a startling piece of news, and clearly demanded the most thorough investigaton, and prompt exposure in the event of the charge being substantiated. A note was at once sent to the proper official of the hospital indicated, calling his attention to these grave charges, and asking him, if possible, to furnish an explanation. The explanation was immediately forthcoming, and with it two original letters—the one from the patient who had attempted suicide, the other from the mother of the patient. These two letters were both addressed to the nurse who was in charge of the would-be suicide, and were written for the purpose of thanking her for the great kindness the patient had received whilst in the hospital. Not only is the nurse most warmly and affectionately thanked, but special reference is made to the very sister who was said to have been so unkind. The following quotation from the patient's letter clearly shows how baseless is the charge:—"I write to thank you for all you have done for me, not only in nursing me but in other ways. You have indeed proved a friend, and I hope you will meet with a just reward. I am sure I must have been a great deal of trouble and anxiety to you in my last illness. I wish so much that I could do something for you to show that I am indeed grateful for all your kindness; also dear Sister

—, will you please thank her for me for all her kindness and patience during my illness?"

Now, it happens that in this case the most complete means of justification are at hand in the shape of these two original letters, which are in our possession; and therefore the charge may be dismissed as baseless and absurd. But how very different it might have been! The poor patient, who was in a melancholy state of mind, twice attempted suicide, but in no sense because of any unkindness on the part of any person in the hospital. Indeed, when one of the attempts was made, the very sister who was charged with the cruelty was herself ill in bed. It is grievous and inexcusable that baseless charges of this kind should be made against persons who, as a class, exhibit a self-denial and a noble devotion to duty which are beyond all praise. And not only is it grievous, but it is foolish, because this charge having been proved untrue, similar charges made in future would be disbelieved, even though they should happen to be true. We are always willing and anxious to hear complaints which may be well founded, but we counsel the public, and especially the lady portion of it, to be very slow in believing charges against institutions so well managed, supervised, criticised, and exposed to the light of day, as are the London hospitals.

